# Neural Tracking in Infancy Predicts Language Development in Children With and Without Family History of Autism

**DOI:** 10.1162/nol_a_00074

**Published:** 2022-08-17

**Authors:** Katharina H. Menn, Emma K. Ward, Ricarda Braukmann, Carlijn van den Boomen, Jan Buitelaar, Sabine Hunnius, Tineke M. Snijders

**Affiliations:** Max Planck Institute for Psycholinguistics, Nijmegen, The Netherlands; Donders Institute for Brain, Cognition and Behaviour, Radboud University, Nijmegen, The Netherlands; Department of Neuropsychology, Max Planck Institute for Human Cognitive and Brain Sciences, Leipzig, Germany; Research Group Language Cycles, Max Planck Institute for Human Cognitive and Brain Sciences, Leipzig, Germany; International Max Planck Research School on Neuroscience of Communication: Function, Structure, and Plasticity, Leipzig, Germany; Department of Experimental Psychology, Helmholtz Institute, Utrecht University, Utrecht, The Netherlands; Department of Cognitive Neuroscience, Radboud University Medical Center, Nijmegen, The Netherlands; Cognitive Neuropsychology Department, Tilburg University

**Keywords:** autism, neural oscillations, speech segmentation, word learning, speech entrainment, speech processing

## Abstract

During speech processing, neural activity in non-autistic adults and infants tracks the speech envelope. Recent research in adults indicates that this neural tracking relates to linguistic knowledge and may be reduced in autism. Such reduced tracking, if present already in infancy, could impede language development. In the current study, we focused on children with a family history of autism, who often show a delay in first language acquisition. We investigated whether differences in tracking of sung nursery rhymes during infancy relate to language development and autism symptoms in childhood. We assessed speech-brain coherence at either 10 or 14 months of age in a total of 22 infants with high likelihood of autism due to family history and 19 infants without family history of autism. We analyzed the relationship between speech-brain coherence in these infants and their vocabulary at 24 months as well as autism symptoms at 36 months. Our results showed significant speech-brain coherence in the 10- and 14-month-old infants. We found no evidence for a relationship between speech-brain coherence and later autism symptoms. Importantly, speech-brain coherence in the stressed syllable rate (1–3 Hz) predicted later vocabulary. Follow-up analyses showed evidence for a relationship between tracking and vocabulary only in 10-month-olds but not in 14-month-olds and indicated possible differences between the likelihood groups. Thus, early tracking of sung nursery rhymes is related to language development in childhood.

## INTRODUCTION

Autistic individuals often experience language difficulties (Eigsti et al., 2011), which usually emerge early in life, with autistic children often showing delays in language acquisition (Howlin, 2003). In non-autistic adults, brain activity synchronizes with incoming speech. This process is referred to as *neural tracking* and is directly linked to language comprehension (Peelle et al., 2013). There are indications that tracking of speech in the theta band is reduced in autistic adults (Jochaut et al., 2015). Reduced tracking may also impact early language development (Goswami, 2019). The current article investigates whether tracking in infancy predicts language acquisition and the development of autism symptoms in children with high and low likelihood for autism.

Autism spectrum disorder is a common neurodevelopmental condition characterized by social communicative differences and restricted repetitive behaviours (American Psychiatric Association, 2013). Our research focuses on the communication aspect, which is often characterized by differences in expressive language as well as language comprehension difficulties. Research suggests that autistic children differ from their non-autistic peers across a broad range of linguistic skills (Kwok et al., 2015), ranging from differences in low-level acoustic speech processing (Cardy et al., 2005; Kasai et al., 2005) to high-level linguistic abstraction such as semantics, syntax, and pragmatics (for reviews, see: Eigsti et al., 2011; Groen et al., 2008). However, the precise nature of these differences varies widely between individuals (Anderson et al., 2007; Groen et al., 2008). Parents often experience a delay or regression of language development as a first sign that their child is not developing typically (Kurita, 1985; Rogers, 2004; Thurm et al., 2014). Howlin (2003) showed that autistic children produce their first word at an average age of 15–38 months, compared to 8–14 months in typically developing children, who were matched for nonverbal IQ.

The exact causes behind language delays in autism remain unknown, but recent evidence indicates they may be related to differences in neural development (Lombardo et al., 2015; Van Rooij et al., 2018; Verly et al., 2014). One hypothesis states that the balance of neural excitation and inhibition (E/I balance) is altered in autistic individuals (Bruining et al., 2020; Dickinson et al., 2016; Rubenstein & Merzenich, 2003; Snijders et al., 2013). This E/I balance is crucial for regulating the flow of information in the brain (Haider et al., 2013; Shew et al., 2011) and also gives rise to neural oscillations (Poil et al., 2012), which underlie a broad range of behavioral, cognitive, and perceptual processes, including language processing (see Meyer, 2018, for an overview). Different development of neural oscillations may thus also affect language development in autistic children. In line with this, recent studies indicate that autistic children show different development in resting-state spectral electroencephalography (EEG) power (Tierney et al., 2012) and that these differences relate to different language development between autistic and non-autistic children (Romeo et al., 2021; Wilkinson et al., 2020).

For assessing neural processing of continuous speech directly, one of the most influential findings in the last years is that adults’ oscillations synchronize with external signals such as speech (Giraud & Poeppel, 2012). The amplitude envelope of speech contains amplitude modulations at different timescales, which to a certain extent correspond to the occurrences of phonemes (30–40 Hz, gamma range), syllables (4–8 Hz, theta range), and intonational phrases (below 4 Hz, delta range). Adults’ neural activity tracks the amplitude modulations of speech in these different frequency bands (Di Liberto et al., 2015; Doelling et al., 2014; Peelle & Davis, 2012), and tracking was shown to be related to language comprehension (Riecke et al., 2018; Vanthornhout et al., 2018). Atypicalities in tracking have been found for language-related neurodevelopmental conditions (Molinaro et al., 2016; Power et al., 2013). To our knowledge, there is currently only one study that focused on speech tracking in autism. Jochaut et al. (2015) examined tracking of continuous speech in 13 autistic adults and 13 non-autistic adults. They found decreased speech tracking for the autistic group compared to the non-autistic group in the theta range (4–7 Hz), which is assumed to synchronize with the typical syllable rate in adult-directed speech. In addition, Jochaut et al. (2015) analyzed individual differences between participants and found a positive correlation between speech tracking and participants’ verbal abilities along with a negative correlation between speech tracking and general autism symptoms. This suggests tracking of speech is related to language processing and possibly also general autism symptoms, but note that this relatively low-sampled study still needs to be replicated.

Atypical tracking may be related to the delay in language acquisition reported for autistic children. One of the first challenges infants need to overcome during language development is segmenting continuous speech into smaller linguistic units, such as words, for language comprehension. Adults rely mostly on linguistic knowledge for speech segmentation (Marslen-Wilson & Welsh, 1978), but infants who still lack the required knowledge need to rely on other cues. To a certain extent, the boundaries of linguistic units are cued by speech acoustics. Leong and Goswami (2015) analyzed the amplitude modulation structure of nursery rhymes, a particularly rhythmic form of infant-directed speech. They found that amplitude modulations were centered around three frequency rates, which match the occurrence rates of stressed syllables (∼2 Hz), syllables (∼5 Hz), and phonemes (∼20 Hz). This means that even infants who still lack linguistic knowledge may be able to extract linguistic units from continuous speech by tracking amplitude modulations (see also Goswami, 2019). Infants with better tracking would thus be at advantage for their initial language acquisition, as they are able to extract and learn the meaning of linguistic units from continuous speech faster. Crucially, the importance of acoustic cues for speech segmentation has been shown to decrease with age, as infants start to use more linguistic knowledge for speech segmentation (Bortfeld et al., 2005; Kidd et al., 2018; Männel & Friederici, 2013). It is unclear when the shift from acoustic to linguistic speech segmentation happens, but both Dutch and English infants have been shown to still rely on prosodic cues for word segmentation at least until 10 months of age (Johnson & Seidl, 2009; Kooijman et al., 2009). Possibly, tracking may be more advantageous for infants earlier in their language development, before they shift towards top-down segmentation strategies. In the current study we compared 10-month-old infants to 14-month-old infants. Between 10 and 14 months, infants show on average a fourfold increase in their receptive vocabulary size (see Frank et al., 2017), indicating the speech segmentation of the 14-month-olds could rely more on linguistic cues. Thus, we assessed whether the importance of tracking specific frequency bands might depend on the infants’ developmental stage. Studies investigating tracking in infants have been rare, but recent results indicate that typically developing infants track the amplitude modulations in speech (Attaheri et al., 2022; Jessen et al., 2019; Kalashnikova et al., 2018; Menn et al., 2022; Ortiz Barajas et al., 2021). It remains unclear, however, how infants’ tracking relates to language development.

The current study investigated the relationship between tracking in infancy, language development, and later autism symptoms. Since autism cannot be reliably diagnosed before the age of three (Charman & Baird, 2002) and the average age of diagnosis is 5 to 7 years (Szatmari et al., 2016), this study employed a prospective longitudinal approach (Bölte et al., 2013; Jones et al., 2019; Loth et al., 2017). We followed younger siblings of autistic children, referred to as high-likelihood siblings as they have a 10–20% likelihood of receiving a later autism diagnosis, compared to a 1% likelihood in the general population (Constantino et al., 2010; Ozonoff et al., 2011). In additon, we also followed a group of infants with an older non-autistic sibling, referred to as low-likelihood group.

We obtained EEG recordings of 10- and 14-month-old infants listening to sung nursery rhymes. Speech-brain coherence to sung nursery rhymes was taken as a measure of tracking. We analyzed tracking of stressed syllables, syllables, and phonemes, since the amplitude modulations of nursery rhymes are particularly pronounced in the corresponding frequency bands (Leong & Goswami, 2015). We then examined the relationship between tracking and behavioral scores of vocabulary at 24 months and autism symptoms at 36 months. Based on findings from autistic adults (Jochaut et al., 2015), we expected a relationship between tracking and both language abilities and autism symptoms. The exact hypotheses for the current experiment were as follows: We expected 10- and 14-month-old infants in the high-likelihood group to show decreased speech-brain coherence compared to the low-likelihood group. On an individual level, we expected speech-brain coherence to correlate with higher vocabulary at age 24 months and lower autism symptoms at age 36 months. Since the importance of acoustic information in the different frequency bands may vary with language development, we also explored the interaction between speech-brain coherence and age for predicting vocabulary development.

## MATERIALS AND METHODS

### Participants

**Figure 1.**
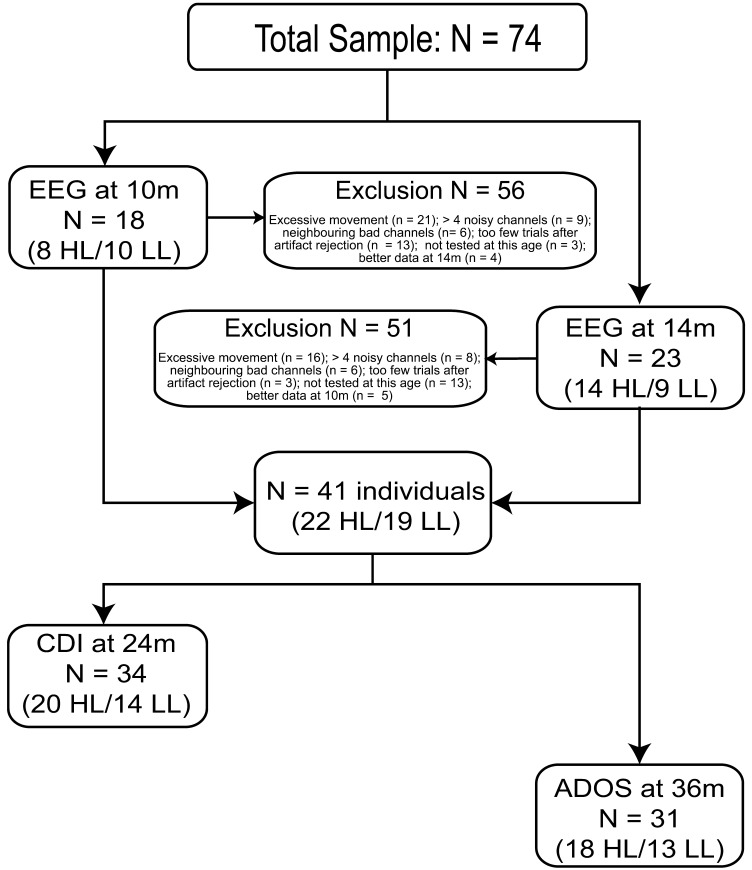
Numbers of infants included in the final analysis. Infants were included if they contributed one usable EEG data set. Our final sample for the first analysis included 22 high-likelihood (HL) infants and 19 low-likelihood (LL) infants. Not all infants provided follow-up measures for vocabulary size (CDI) or autism symptoms (ADOS).

All participants of this study were tested within a broader project investigating the early development of autism (Jones et al., 2019). For this study, we obtained the data of 74 Dutch infants: 45 high-likelihood infants and 29 low-likelihood infants. High-likelihood infants (HL) had an older autistic sibling, and low-likelihood infants (LL) had an older non-autistic sibling and no family history of autism, psychiatric, or genetic conditions. All infants were raised in the Netherlands and tested at one of two testing sites. Forty-seven of the infants (30 HL, 17 LL) were tested in the infant laboratory at site 1, the other 27 (15 HL, 12 LL) were tested at their homes by researchers from site 2. For the at-home tests, experimenters took care to create a homogeneous and non-distracting environment by placing a tent on the table that surrounded the child and screen. As such, the visual environment was similar for all children (see, e.g., Di Lorenzo et al., 2019). Infants were included in the final analysis if they provided one usable EEG data set. Exclusion criteria were excessive movement during testing, more than four noisy channels, neighboring bad channels, or failure to reach the minimum trial criterion after artifact rejection. Figure 1 displays the final sample of infants after exclusion, as well as the number and reasons for exclusions per age point. Since only 9 infants provided usable EEG data for both age points, we decided to use only one EEG data set per infant. The final sample included a total of 41 infants with one usable EEG data set (22 HL, 19 LL). Thirty-four of these infants also had vocabulary scores at 24 months available (20 HL, 14 LL), and 31 had autism measures at 36 months (18 HL, 13 LL). Table 1 summarizes the descriptive statistics per testing. The experimental procedure was approved by the relevant ethics committee at each site and was conducted in accordance with the Declaration of Helsinki.

**Table 1.** Demographics of the children included in the final analysis per testing

Likelihood	**EEG: 10 months**			**EEG: 14 months**			**CDI: 24 months**			**ADOS: 36 months**		
	*N*	Age (*SD*)	Sex (f:m)	*N*	Age	Sex	*N*	Age	Sex	*N*	Age	Sex
HL	8	10,26 (0, 72)	5:3	14	14,06 (0, 5)	8:6	20	24,7 (1)	13:7	18	38,8 (5)	13:5
LL	10	10 (0, 6)	4:6	9	14,45 (0, 6)	4:5	14	24,8 (1, 3)	6:8	13	38,4 (3)	6:7

*Note*. HL: High-likelihood infants. LL: Low-likelihood infants. SDCDI: Vocabulary size. ADOS: Autism symptoms.

### Materials

#### Stimuli

The stimuli consisted of five sung nursery rhymes that are highly familiar to Dutch infants (Jones et al., 2019): “Dit zijn mijn wangetjes” (translation: These are my cheeks; duration: 16.4 s), “De wielen van de bus” (Wheels on the bus; 12.5 s), “Hansje pansje kevertje” (Hansje pansje beetle; 10.6 s), “Twinkel twinkel kleine ster” (Twinkle twinkle little star; 13 s), “Papegaaitje leef je nog?” (Parrot are you still alive?; 17 s). Video recordings were made of two female native Dutch speakers, alternately singing the nursery rhymes. Speakers were instructed to present the nursery rhymes in an infant-directed manner, while making accompanying gestures. The total duration of the video recordings was 69 seconds. To identify the most important amplitude modulation frequencies in the speech envelope in our stimuli, we transcribed the duration of all stressed syllables, syllables and phonemes using Praat (Boersma, 2001). In our stimuli, 85% of all stressed syllables occurred at a rate of 1–3 Hz and 85% of all phonemes occurred at a rate between 5 and 15 Hz. In addition, we also looked at infants’ tracking in the frequency rate from 3 to 5 Hz, which mostly captures the syllables. Note that 85% of all syllables in the stimuli occurred within 1.7–6 Hz, but we limited the syllable rate to 3–5 Hz to avoid overlap with the stressed syllable and phonological rate. We put more emphasis on stressed syllables and phonemes, as these acoustic-phonological cues are thought to be especially relevant for infant language acquisition (Gervain & Mehler, 2010). These frequency rates used in this study are slower than the frequency rates typically analyzed in adult studies, including the study by Jochaut et al. (2015), but are similar to the modulation rate previously reported for infant-directed speech (Leong et al., 2017), nursery rhymes (Leong & Goswami, 2015), and songs (Ding et al., 2017).

#### Behavioral tests

The vocabulary knowledge of the children was tested using the Dutch version of the MacArthur-Bates Communicative Development Inventories (CDI), a standardized vocabulary test for children between 10 months and 36 months. It is a parent report measure of both receptive and productive vocabulary with high reliability (Zink & Lejaegere, 2002). The CDI was filled in by one of the child’s caregivers when the child was approximately 24 months old. To account for variability in children’s age at administration, the test scores of receptive and productive vocabulary were transformed to age-normed percentile scores.

Autism symptoms were measured using the Autism Diagnostic Observation Schedule-Second Edition (ADOS-2; Lord et al., 2000). The ADOS-2 is a highly reliable and valid measure for autistic symptoms (Bölte & Poustka, 2004). Depending on the linguistic ability of the child, Module 1 or Module 2 of the test was administered by a trained psychologist. For our analyses, we used the comparison scores, which allow a reliable comparison of performance on the different modules. The scores range from 1 to 10, with scores from 4 to 7 suggesting medium indication for autism and scores of 8 or more suggesting high indications for autism.

### Procedure

During the EEG recordings, infants sat either on their parent’s lap or in a highchair in front of a computer screen with approximately 1 m distance to the screen (24 inch, 16:9, 1920 × 1080 pixels) on which the stimuli were presented. The nursery rhymes were presented three times during a session, leading to a total duration of 207 seconds. They were shown as part of a larger experiment intermixed with other experimental conditions. The total experiment took about 20 minutes during which EEG was recorded continuously.

### EEG Recordings

At site 1 a 32-channel actiCAP system by Brain Products was used. Site 2 made use of a 32-channel active electrode set by Biosemi. The main differences between the recordings of the two systems are: different placement for four electrodes (Biosemi: AF3, AF4, PO3, PO4 vs. actiCAP: TP9, TP10, PO9, PO10), a different sampling rate (Biosemi: 2048 Hz, actiCAP: 500 Hz), and different online reference electrodes (Biosmi: CMS and DLR electrodes, actiCAP: AFz). The final analysis included only electrodes measured on both sites, namely: FP1/2, Fz, F3/4, F7/8, FC1/2, FC5/6, Cz, C3/4, T7/8, CP1/2, CP5/6, Pz, P3/4, P7/8, Oz, O1/2.

#### EEG pre-processing

The EEG analysis was performed using the Fieldtrip toolbox (Oostenveld et al., 2011) in Matlab R2016a. To accommodate for the differences in recording systems, Biosemi data were first down-sampled to 500 Hz and re-referenced to Cz. To improve the independent component analysis (ICA) and channel interpolation, we reduced the electrodes to the final subset only after preprocessing.

As a first pre-processing step, data were high-pass filtered at 0.1 Hz and low-pass filtered at 45 Hz. Next, we performed ICA on the whole data set to remove noise by ocular movements or noisy electrodes. We identified on average 1.8 (range: 0–6) noise components per data set. Afterwards, the electrophysiological data corresponding to the presentation of nursery rhymes were extracted from the data set and divided into 3 s epochs using a sliding window with two thirds overlap. This led to a maximum of 201 epochs per infant. ICA components capturing noise were removed from the epochs and a maximum of four non-neighbouring channels per infant were repaired using a spline interpolation (Perrin et al., 1989). The 28 final electrodes were rereferenced to the common average of all electrodes. Finally, epochs were demeaned and all EEG epochs containing fluctuations ±150 *μ*V were excluded using automatic artifact rejection. Only infants with at least 30 artifact-free epochs were included in the final analysis. Since only 9 infants provided usable EEG data for both age points, we decided to use only one EEG data set per infant. Per infant, we included the data set with more artifact-free epochs, either from 10 months (*n* = 18) or from 14 months (*n* = 23), in our final analysis. On average, infants contributed 98 artifact-free epochs to the analysis.

### Analysis

#### Speech-brain coherence

Speech-brain coherence was established by first computing the speech envelope of the stimuli using a Hilbert transform with a 4th-order Butterworth filter. Then, we took the Fourier transform of both the speech envelope and the EEG data from 1 to 15 Hz (with a frequency resolution of 0.33 Hz), which corresponds to the most important linguistic properties in our stimuli. Coherence was computed as the cross-spectrum between EEG electrode signal *x* and speech signal *y*, normalized by the power spectra of these signals (Rosenberg et al., 1989).

$${Coh}_{xy}=\frac{\left|\langle S_{xy}\rangle \right|}{\sqrt{\langle S_{xx}\rangle *\langle S_{yy}\rangle }}$$

The coherence values reflect the consistency of the phase difference between the two signals at a given frequency. Importantly, this means that we directly look at the synchronization between speech and brain activity (a similar approach has been used in Peelle et al., 2013).

To analyze the presence of speech-brain coherence, we compared the observed speech-brain coherence to surrogate data. This was computed by shuffling the speech envelope across epochs and computing the average coherence over 100 pairings of a random speech envelope with the EEG data. We then used a cluster-based permutation test to analyze the coherence difference between the observed and the surrogate data in the frequency range from 1 to 15 Hz, allowing us to assess all frequencies within one single test (Maris & Oostenveld, 2007).

#### Relationship speech-brain coherence with behavior

The relationship between speech-brain coherence and the behavioral measures was analyzed in R 3.5.1 (R Core Team, 2018) with RStudio 1.1.456 (RStudio Team, 2016). All graphs were created using the ggplot (Wickham, 2016) and the gghalves (Tiedemann, 2020) packages.

For the analysis, we first normalized the coherence values to ensure that different numbers of trials per child did not influence our result (see Bastos & Schoffelen, 2016). For normalization, we used the following formula:

$${\text{Coherence}}_{\text{normalized}}=\frac{{\text{Coherence}}_{\text{observed}}-{\text{Coherence}}_{\text{surrogate}}}{{\text{Coherence}}_{\text{observed}}+{\text{Coherence}}_{\text{surrogate}}}$$

We then averaged the normalized coherence values across all electrodes within the three frequency bands of interest: The stressed syllable rate (1–3 Hz), the syllable rate (3–5 Hz), and the phonological rate (5–15 Hz), leading to one coherence value per frequency band per infant.

To test for a group difference between HL and LL infants, we first ran a repeated-measures analysis of variance (ANOVA) using coherence as dependent variable, frequency band (stressed syllable/syllable/phonological) as within-subjects factor, and likelihood group (low/high) and age group (10 m/14 m) as between-subject factors.

To test for a relationship between coherence and behavior, we ran separate linear regression models using the receptive vocabulary percentile on the CDI, the productive vocabulary percentile on the CDI, and the comparison scores of the ADOS as dependent variables. Since the range of autism symptoms in the LL group was very low (see Figure 5A), the last model was only run in the HL group. Because the coherence measures across the different frequency bands are correlated, we entered the predictors in three steps for each regression model. Given the limited research on speech tracking in infancy, we entered the coherence rates in order of the importance of the different acoustic cues for language development. In the first step, we added: Coherence in the stressed syllable rate, the interaction between coherence and age group, and the interaction between coherence and likelihood group (only for the language models). We first entered coherence in the stressed syllable rate, since prior research established a relationship between word segmentation of trochaic words and vocabulary development (Junge et al., 2012; Jusczyk, 1999). In the second step, we added coherence in the phonological rate, and its interactions with both age group and likelihood group. Prior research established a relationship between phonetic perception and language development (Kuhl et al., 2008). In the third step, coherence in the syllable rate as well as its interactions with age group and likelihood group were added to the model. Models were compared using the ANOVA function and new predictors were only retained if they significantly improved the model fit. In addition, we used the caret package (Kuhn, 2008) to perform Monte Carlo cross-validation (with 200 repetitions, each holding back 20% of the sample) and assess the generalizability of the regression models (de Rooij & Weeda, 2020; Song et al., 2021). For follow-up analyses yielding significant effects on the group level we used leave-one-out cross-validation to account for the small group sizes.

## RESULTS

### Speech-Brain Coherence

Speech-brain coherence was significantly higher for the observed data than for the surrogate data (*p* < 0.001). In the cluster-based permutation analysis, one large cluster emerged that included all electrodes in the frequencies from 1 to 15 Hz, covering the phonological, syllable, and stressed syllable ranges. This indicates that across the groups, infants showed tracking of sung nursery rhymes.

### Relationship Speech-Brain Coherence and Behavior

#### Group differences

**Figure 2.**
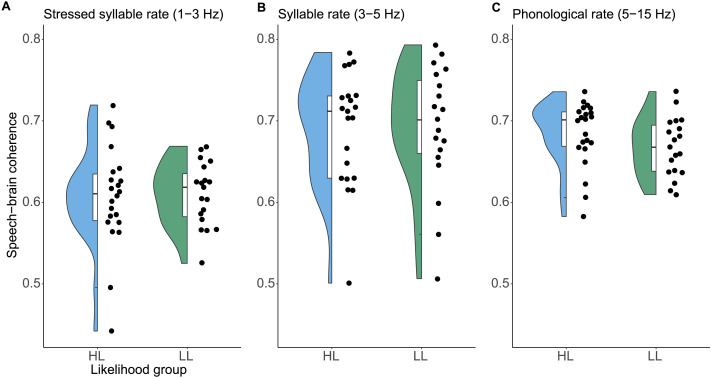
Coherence values for the HL and the LL group in (A) the stressed syllable rate (1–3 Hz), (B) the syllable rate (3–5 Hz), and (C) the phonological rate (5–15 Hz). Dots depict individual data points.

Speech-brain coherence in the HL group did not significantly differ from speech-brain coherence in the LL group. The repeated-measures ANOVA showed no significant main effect of likelihood group, *F*(1, 37) = 0.22, *p* = 0.6385, and age group, *F*(1, 37) = 0.002, *p* = 0.9626, and no significant interactions, all *F*s < 0.36. There was a significant main effect of frequency rate, *F*(2, 74) = 26.36, *p* < 0.0001, indicating that mean coherence values differed between the frequency rates. Follow-up *t* tests showed that normalized coherence in the stressed syllable rate (*M* = 0.61, *SD* = 0.05) was significantly lower compared to the syllable rate (*M* = 0.69, *SD* = 0.07), *t*(40) = −5.83, *p* < 0.0001, and the phonological rate (*M* = 0.66, *SD* = 0.04), *t*(40) = −9.23, *p* < 0.0001. The syllable and the phonological rate did not significantly differ, *t*(40) = 1.31, *p* = 0.199. Figure 2 shows the distribution of coherence scores in the frequencies of interest for both likelihood groups separately.

#### Vocabulary

**Figure 3.**
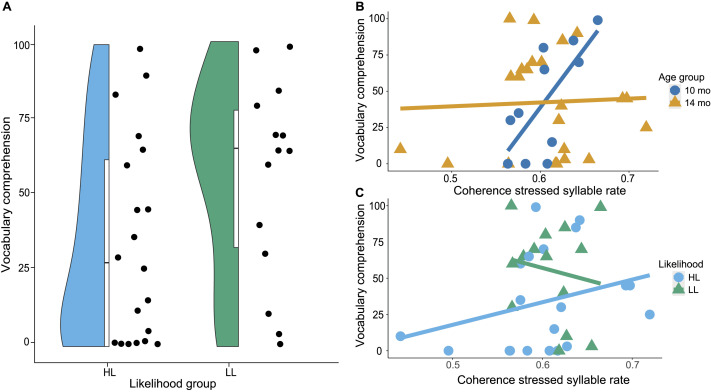
Relationship between coherence in infancy and receptive vocabulary in childhood. (A) Distribution of CDI receptive vocabulary percentiles for both likelihood groups. (B) Relationship between receptive vocabulary on the CDI at 24 months and speech-brain coherence in the stressed syllable rate (1–3 Hz) by age group. (C) Relationship between speech-brain coherence in the stressed syllable rate and receptive vocabulary by likelihood group.

Figure 3A shows the distribution of CDI percentile scores for receptive vocabulary for both likelihood groups. Descriptively, the LL group had higher receptive vocabulary (*M* = 55.5, *SD* = 33.7) than the HL group (*M* = 33.85, *SD* = 34). This difference was not statistically significant, *t*(32) = 1.83, *p* = 0.076. Results of the first step of the linear regression indicated a significant model fit, *F*(3, 30) = 4.6, *p* = 0.0091, *R*_*CV*_^2^ = 0.41, *RMSE*_*CV*_ = 28.84. Further examination of the individual predictors showed that receptive vocabulary was significantly predicted by coherence in the stressed syllable rate, *t* = 3.65, *p* < 0.001, the interaction between coherence in the stressed syllable rate and age group, *t* = −3.33, *p* = 0.0023, and the interaction between coherence in the stressed syllable rate and likelihood group, *t* = −2.47, *p* = 0.0195. Figures 3B–C present the data for the relationship between receptive vocabulary and speech-brain coherence split by age group and likelihood group, respectively. Post hoc analyses showed the correlation was significant for the 10-month-olds, *r*(9) = 0.71, *p* = 0.0134, *R*_*CV*_^2^ = 0.349, *RMSE*_*CV*_ = 29.22, but not the 14-month-olds, *r*(21) = 0.05, *p* = 0.834. The correlation for the likelihood groups were both non-significant (LL: *r*(12) = −0.16, *p* = 0.5783; HL: *r*(18) = 0.29, *p* = 0.2117). There was one outlier in the HL group. Removal of this value did not change the pattern of results so we decided to include it in the analyses reported here. In the second step of the model, inclusion of phonological coherence and its interactions with age and likelihood group did not significantly improve the fit of the model, *F*(3, 27) = 0.75, *p* = 0.5333, and had lower generalizability, *R*_*CV*_^2^ = 0.28, *RMSE*_*CV*_ = 33.12. Coherence in the phonological rate was not predictive of receptive vocabulary, *t* = 1.03, *p* = 0.3108, nor was the interaction between phonological rate and age group, *t* = −1.46, *p* = 0.1557, or likelihood group, *t* = 0.15, *p* = 0.8785. Since the second model did not significantly improve the fit over the first model, we compared the fit of the third model in the next step to the first model again. Model comparisons showed that the addition of coherence in the syllable rate and its interactions with age and likelihood group did not significantly improve the model fit, *F*(3, 24) = 0.59, *p* = 0.6288, and decreased model generalizability, *R*_*CV*_^2^ = 0.27, *RMSE*_*CV*_ = 32.65. Inspection of the individual predictor terms found no significant effect of coherence in the syllable rate on receptive vocabulary, *t* = −0.05, *p* = 0.9627, nor of its interactions with age group, *t* = −0.42, *p* = 0.6756, or likelihood group, *t* = −0.37, *p* = 0.7145. The results indicate a relationship between coherence specifically in the stressed syllable range (1–3 Hz) and the development of receptive vocabulary. The interactions indicate that coherence in the stressed syllable rate was a predictor for receptive vocabulary for 10-month-olds but possibly not for 14-month-olds (see Figure 3B). In addition, the relationship between tracking in the stressed syllable rate and perceptive vocabulary was possibly stronger in the high-likelihood group compared to the low-likelihood group (see Figure 3C), but note that the post hoc tests were not significant in either group.

**Figure 4.**
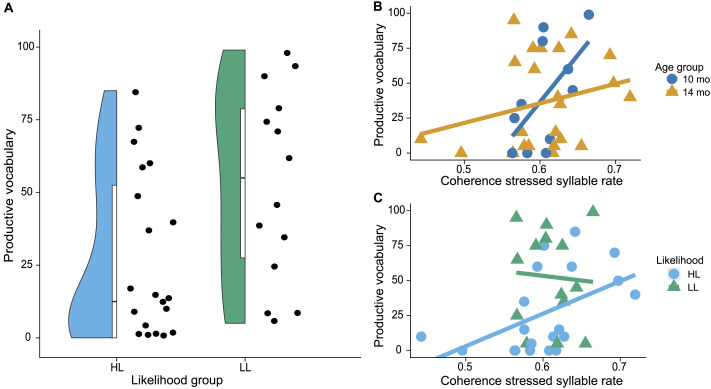
Relationship between coherence in infancy and productive vocabulary in childhood. (A) Distribution of CDI productive vocabulary percentiles for both likelihood groups. (B) Relationship between speech-brain coherence and productive vocabulary by age group. (C) Relationship between speech-brain coherence and productive vocabulary by likelihood group.

For productive vocabulary, the results were similar to those for receptive vocabulary, as depicted in Figure 4. Productive vocabulary was significantly higher in the LL group (*M*_*LL*_ = 57.79, *SD* = 34.35) than in the HL group (*M*_*HL*_ = 27, *SD* = 29.44), *t*(32) = 2.35, *p* = 0.0253. The first step of the regression showed a significant model fit, *F*(3, 30) = 3.6, *p* = 0.0247, *R*_*CV*_^2^ = 0.292, *RMSE*_*CV*_ = 30.51. Inspection of the individual predictors showed that coherence in the stressed syllable rate was a significant predictor of productive vocabulary, *t* = 2.97, *p* = 0.0059. In addition, we found a significant interaction between coherence in the stressed syllable rate and age group, *t* = −2.36, *p* = 0.0248, and the interaction between coherence in the stressed syllable rate and likelihood group trended toward significance, *t* = −1.98, *p* = 0.0568. Post hoc analyses showed that the correlation was significant for the high-likelihood group, *r*(18) = 0.50, *p* = 0.0235, but had a low generalizability *R*_*CV*_^2^ = 0.02, *RMSE*_*CV*_ = 28.45, and was not significant for the low-likelihood group, *r*(12) = −0.06, *p* = 0.8276. The correlation approached significance for the 10-month-olds, *r*(9) = 0.59, *p* = 0.058, *R*_*CV*_^2^ = 0.2, *RMSE*_*CV*_ = 33.44, and was not significant for the 14-month-olds, *r*(21) = 0.26, *p* = 0.2298. Inclusion of coherence in the phonological rate and its interactions with age and likelihood group did not significantly improve model fit, *F*(3, 27) = 0.88, *p* = 0.4623, and decreased generalizability, *R*_*CV*_^2^ = 0.27, *RMSE*_*CV*_ = 34.16. Inspection of the new predictors in the second step showed that neither coherence in the phonological rate, *t* = 0.83, *p* = 0.4114, nor its interactions with age, *t* = −0.74, *p* = 0.4643, or likelihood group, *t* = −1.31, *p* = 0.2016, significantly predicted productive vocabulary. The inclusion of coherence in the syllable rate and its interactions with age group and likelihood group in the third step did not significantly improve model fit compared to the first model, *F*(3, 27) = 1.02, *p* = 0.4004, and led to a lower generalizability, *R*_*CV*_^2^ = 0.23, *RMSE*_*CV*_ = 35.1. Inspection of the individual new predictors did not show a significant effect of coherence in the syllable rate on productive vocabulary, *t* = −1.29, *p* = 0.2097, nor a significant interaction of coherence in the syllable rate with age group, *t* = 0.33, *p* = 0.7427, or likelihood group, *t* = 1.13, *p* = 0.2696.

Note we always assessed the average of the speech-brain-coherence across electrodes to increase power. For exploratory purposes, topographic maps displaying the correlations between stressed syllable speech-brain coherence and vocabulary are shown in Figure S1 (Supporting Information can be found at https://doi.org/10.1162/nol_a_00074). As we included stressed syllable rate first, it might be that the other rates are explaining the same variance, but no additional variance, and because of that they turned out to be non-significant predictors. To check for this possibility, we ran models predicting receptive and productive vocabulary including only phonological rate or only syllable rate and their respective interactions with age and likelihood group as predictors. The models did not reach significance, all *p*s > 0.157, suggesting that the identified relationships with vocabulary were indeed specific to the stressed syllable rate.

#### Autism symptoms

**Figure 5.**
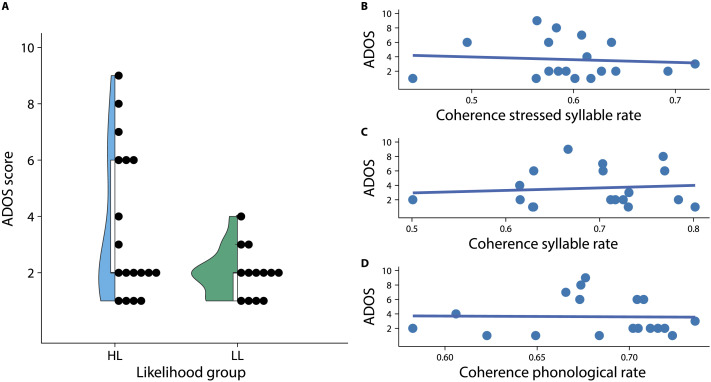
Relationship between coherence in infancy and autism symptoms in childhood. (A) presents the distribution of ADOS scores in the HL and the LL groups. (B) shows the data for the relationship between speech-brain coherence in the stressed syllable rate (1–3 Hz) and the ADOS score for the HL group. (C) shows the relationship between speech-brain coherence in the syllable rate (3–5 Hz) and the ADOS score. (D) shows the relationship between speech-brain coherence in the phonological rate (5–15 Hz) and the ADOS score.

Figure 5A depicts the distribution of ADOS scores for both likelihood groups. We only tested the relation between ADOS scores and speech-brain coherence in the HL group. The model fit for the first model predicting ADOS scores was not significant, *F*(2, 15) = 0.06, *p* = 0.9394. Inspection of the individual predictors showed no significant main effect of coherence in the stressed syllable rate, *t* = −0.01, *p* = 0.9891, and no interaction between coherence in the stressed syllable rate and age group, *t* = −0.08, *p* = 0.9402. The inclusion of phonological coherence, *t* = 0.22, *p* = 0.8298, and its interaction with age group, *t* = −0.206, *p* = 0.8398, did not significantly improve the model fit, *F*(2, 13) = 0.02, *p* = 0.9759. In the third step, adding coherence in the syllable rate, *t* = 1.3, *p* = 0.2165, and its interaction with age group, *t* = −1.32, *p* = 0.2107, did not improve model fit compared to the first step, *F*(2, 13) = 0.91, *p* = 0.4253. The relationship between coherence in the different frequency rates and ADOS scores is depicted in Figure 5B–D.

## DISCUSSION

The current study investigated the relationship between neural tracking in infancy and development of vocabulary and autism symptoms in early childhood. We expected that infants with a high likelihood for autism would show decreased speech-brain coherence compared to a low-likelihood comparison group. In addition, we expected that increased speech-brain coherence in infancy would be related to better receptive and productive vocabulary at 24 months and fewer autism symptoms at 36 months.

We identified speech-brain coherence to sung nursery rhymes in infants. Overall, infants showed more coherence between the speech envelope and EEG data than expected by chance across all tested frequencies (1–15 Hz) and electrodes. Speech-brain coherence to our sung nursery rhymes might be larger than if we had used spoken stimuli, as results from Vanden Bosch der Nederlanden et al. (2020) suggest that the regular rhythm of songs can aid phase-locking compared to speech.

We found no evidence for a difference in speech-brain coherence between the HL and LL groups and no support for a relationship between speech-brain coherence and the later ADOS score in the HL group. Importantly, we did observe a significant relationship between speech-brain coherence and later vocabulary development. Infants with higher speech-brain coherence in the stressed syllable rate showed higher receptive and productive vocabulary. Follow-up correlation analyses only showed evidence for this effect in the 10-month-old group but no evidence for such an effect in the 14-month-old group. The relationship between coherence and vocabulary also seemed to be stronger for the high-likelihood group compared to the low-likelihood group, but this should be interpreted with care, as follow-up correlations were non-significant for both groups.

Tentatively, the relationship between tracking of stressed syllables and vocabulary might be based on individual differences in infants’ word segmentation skills, which then predict later vocabulary development (Junge et al., 2012; Kooijman et al., 2013). In stress-based languages like English or Dutch, stressed syllables can provide a valuable cue for segmenting words from continuous speech (Jusczyk, 1999), as the majority of content words in these languages have word-initial stress (Cutler & Carter, 1987; Stärk et al., 2021). This effect may be even stronger in infant-directed speech, as caregivers increase amplitude modulations in the prosodic stress rate when addressing infants (Leong et al., 2017) and it was shown that infants’ tracking is sensitive to this adaptation (Menn et al., 2022). High speech-brain coherence indicates an alignment between peaks in neural activity and relevant input (Schroeder & Lakatos, 2009) such as stressed syllables and may thus aid or reflect word segmentation. This idea is supported by a recent study showing a relation between infants’ speech-brain coherence at the stressed syllable rate and word-segmentation performance (Snijders, 2020). In the current study, we provide evidence for a long-term relationship between higher tracking in infancy and vocabulary development.

While acoustic cues may be initially beneficial for speech segmentation, listeners must also use different cues for word segmentation, as there is no perfect relationship between acoustic and linguistic units. Research has shown that adults employ linguistic knowledge, most importantly lexical knowledge, for top-down word segmentation (Cole & Jakimik, 1980; Marslen-Wilson & Welsh, 1978). This indicates that there is a transition from bottom-up to top-down word segmentation during language development, as linguistic knowledge increases (Kidd et al., 2018). There are some indications that lexical knowledge can top-down influence tracking, at least for artificial language learning. For example, Choi et al. (2020) tested infants in a statistical learning paradigm in which they presented 6-month-olds with trisyllabic pseudowords concatenated to syllable strings. While infants initially phase-locked to the syllable rate, they progressed to phase-locking to the trisyllabic word rate over the course of the familiarization phase. A transition from bottom-up to top-down word segmentation could explain the interaction between age and speech-brain coherence in the stressed syllable rate for predicting vocabulary development, as observed in the current study. Bottom-up word segmentation based on acoustic cues may still be beneficial for 10-month-olds, who do not yet have much lexical knowledge, and stronger tracking at this age predicts larger later vocabulary. On the other hand, 14-month-olds have acquired more lexical knowledge and may thus shift from bottom-up to top-down word segmentation of continuous speech. Higher speech-brain coherence would therefore indicate better word segmentation and later vocabulary development in the younger age group, but not in the older age group. Note that at this point this interpretation is rather speculative and needs to be corroborated in the future. Also keep in mind that the final included sample to assess the relationship with vocabulary was rather small (11 10-month-olds), so replication is necessary.

However, following this explanation, it may be the case that infants who are delayed in their language development also transition later from bottom-up to top-down word segmentation. Such a delay could explain the interaction between likelihood group and tracking in the stressed syllable rate for predicting vocabulary knowledge. If the low-likelihood group transitions from bottom-up to top-down speech segmentation earlier, tracking of the stressed syllable rate could be more predictive of their vocabulary development at 10 months and less predictive at 14 months of age. For the high-likelihood group, a later transition would mean that tracking in the stressed syllable rate stays predictive for their vocabulary development longer. It is also possible that autistic children focus more on acoustic cues in general. In line with this, Pomper et al. (2021) showed that autistic toddlers rely more on coarticulation cues during lexical processing than non-autistic toddlers. Both of these explanations are rather speculative at this moment, as our sample size did not allow us to test for a three-way interaction between likelihood group, age group, and speech-brain coherence. It is also possible that the interaction between likelihood group and speech-brain coherence in the stressed syllable rate is based on higher heterogeneity in vocabulary scores in the high-likelihood group.

The relationship between tracking in the stressed syllable rate and vocabulary development may also be explained by other factors than differential use of acoustic cues, such as differences in audiovisual speech processing or selective attention. Infants start to integrate visual information concurrent with speech at an early age (Rosenblum et al., 1997), and better audiovisual integration in infancy predicts better language development (Kushnerenko et al., 2013). In addition, infants with an older autistic sibling show decreased audiovisual integration (Guiraud et al., 2012). Such differences in audiovisual integration of speech information may also affect neural tracking of speech. Past research has shown that visual information increases speech tracking (Crosse et al., 2015; Golumbic et al., 2013; Power et al., 2013), either by enhancing acoustic processing itself or by providing additional information the brain tracks such as the rhythm of lip movements (Bourguignon et al., 2020; Park et al., 2016, 2018). The facilitation of tracking by visual information was shown to be especially strong in preverbal infants (Tan et al., 2022). Since the current study presented the nursery rhymes as videos, which included gestures and other facial information of the speaker during the presentation, we cannot exclude the possibility that differences in audiovisual integration between infants may have contributed to our findings. Another possibility is that we measured differences in attentional resources. Neural tracking is affected by attention (Fuglsang et al., 2017) and reflects the selection of relevant attended information (Obleser & Kayser, 2019). It is thus possible that the relationship between tracking in the stressed syllable rate and later vocabulary reflects individual differences in general attention abilities between the infants. Tentative evidence for this comes from the fact that infants’ attention to speech as well as specifically to lexical stress predicts later vocabulary (Ference & Curtin, 2013; Vouloumanos & Curtin, 2014). Future research should specify how the use of video affects infants’ speech-brain coherence compared to audio-only stimuli and how speech-brain coherence in infants is affected by selective attention.

Contrary to our predictions, we did not find evidence for a relationship between tracking of sung nursery rhymes in infancy and autism symptoms. This is surprising, given that autistic children often have language impairments (Belteki et al., 2022) and we find a relationship between tracking and language development. One reason could be, that speech-brain coherence only captures the language component of autism symptoms, whereas the ADOS captures a broad range of autism symptoms. Tracking of speech might be more sensitive to the development of language specific impairments than to general autism symptoms.

Nevertheless, the data of this developmental study is not in line with the findings by Jochaut et al. (2015), who find a relationship between speech tracking and ADOS scores in their sample of 13 autistic adults. This discrepancy could be explained in different ways. First of all, the null effect could be caused by low power. Despite large variability in ADOS scores, our final analysis included only six children with indications of autism and two who met the diagnostic criterion of autism on the ADOS. This sample might be too small to find a relationship, especially if the relationship shows a similar age-related modulation as we observed for language development. The relationship between tracking and autism symptoms might emerge in a bigger data set with more children who meet the diagnostic criteria for autism. A second possible explanation is that the two groups may have differed in their tracking of spoken stimuli, but that the song modality used in the current study provides additional prosodic cues that make it easier for the HL group to track (Audibert & Falk, 2018; Vanden Bosch der Nederlanden et al., 2020). Thirdly, it is possible that the difference in tracking in autistic individuals only emerges after infancy. During childhood, there are still many developmental changes that affect neural oscillations (Maguire & Abel, 2013), and autism has been linked to differences in the development of key brain structures and neurotransmitters during childhood and adolescence (Courchesne et al., 2007; Van Rooij et al., 2018). Changes in tracking could thus still emerge after infancy. A fourth possible explanation for the difference with the findings by Jochaut et al. (2015) is that the ADOS score might primarily be related to the interactions between different oscillatory frequencies (Arnal & Giraud, 2012). During oscillatory nesting, lower-frequency oscillations influence the amplitude of higher-frequency oscillations. While Jochaut et al. (2015) found a difference for tracking in the theta band between autistic and non-autistic adults, individual measures of autism symptoms were related to an atypical interaction between theta and gamma oscillations. The limited data available in our study did not allow us to precisely replicate this analysis (Tort et al., 2010).

While we saw a developmental pattern in the relationship between tracking and language acquisition, our cross-sectional analysis makes it difficult to draw conclusions about the temporal development of tracking during infancy. Future studies should focus on the individual development of tracking, both in younger age groups (while bottom-up segmentation strategies are still developing) and as children acquire more linguistic knowledge. Furthermore, it would be very interesting to investigate how within-subject changes in tracking during infancy predict later language development. Such research could further test the theory that infants transition from using bottom-up cues to top-down cues for word segmentation from continuous speech. The current study contributes an empirical foundation for such future investigations, by relating tracking in infancy to language development in early childhood but also showing that this relationship might depend on age and linguistic ability.

### Conclusion

This study focused on neural tracking of sung nursery rhymes in infancy and its relationship to the development of vocabulary and autism symptoms in childhood. We analyzed a data set of infants with high- and low-likelihood for autism. With this study, we replicate earlier studies indicating that infants’ neural activity tracks speech. Most importantly, we show that tracking of nursery rhymes during infancy is predictive for later vocabulary development. This finding sheds new light on the importance of oscillatory brain activity in infancy for first language acquisition.

## ACKNOWLEDGMENTS

The authors would like to thank all the families who participated in this research, as well as Loes Vinkenvleugel and Yvette De Bruijn for their assistance with running the project, and Lars Meyer for his valuable feedback on an earlier version of this manuscript. This work has been supported by the EU-AIMS (European Autism Interventions) and AIMS-2-TRIALS programmes, which receive support from Innovative Medicines Initiative Joint Undertaking Grant No. 115300 and 777394, the resources of which are composed of financial contributions from the European Union’s FP7 and Horizon 2020 Programmes, from the European Federation of Pharmaceutical Industries and Associations (EFPIA) companies’ in-kind contributions, and from AUTISM SPEAKS, Autistica and SFARI; by the Horizon 2020 supported programme CANDY Grant No. 847818; and by the Horizon 2020 Marie Sklodowska-Curie Innovative Training Network 642996, BRAINVIEW. The funders had no role in the design of the study; in the collection, analyses, or interpretation of data; in the writing of the manuscript, or in the decision to publish the results. Any views expressed are those of the author(s) and not necessarily those of the funders.

## FUNDING INFORMATION

Jan Buitelaar, Innovative Medicines Initiative Joint Undertaking, Award ID: 115300. Jan Buitelaar and Sabine Hunnius, Innovative Medicines Initiative Joint Undertaking, Award ID: 77394. Jan Buitelaar and Sabine Hunnius, Horizon 2020 Marie Sklodowska-Curie Innovative Training Network, Award ID: 642996. Jan Buitelaar and Sabine Hunnius, Horizon 2020 CANDY, Award ID: 847818.

## AUTHOR CONTRIBUTIONS

**Katharina H. Menn**: Conceptualization: Equal; Formal analysis: Lead; Software: Equal; Visualization: Lead; Writing – original draft: Lead; Writing – review & editing: Lead. **Emma K. Ward**: Data curation: Equal; Investigation: Equal; Project administration: Equal; Software: Supporting; Writing – review & editing: Equal. **Ricarda Braukmann**: Investigation: Equal; Project administration: Equal; Software: Equal. **Carlijn van den Boomen**: Data curation: Equal; Investigation: Equal; Project administration: Equal; Resources: Equal; Software: Equal; Writing – review & editing: Equal. **Jan Buitelaar**: Conceptualization: Supporting; Funding acquisition: Lead; Resources: Equal; Supervision: Supporting; Writing – review & editing: Equal. **Sabine Hunnius**: Conceptualization: Supporting; Funding acquisition: Equal; Resources: Equal; Supervision: Supporting; Writing – review & editing: Equal. **Tineke M. Snijders**: Conceptualization: Lead; Formal analysis: Equal; Resources: Equal; Software: Equal; Supervision: Lead; Writing – original draft: Equal; Writing – review & editing: Lead.
