# Supplementary material for: Neural Tracking in Infancy Predicts Language Development in Children With and Without Family History of Autism
Source: Neurobiol Lang (Camb). 2022 Aug 17;3(3):495–514. doi: 10.1162/nol_a_00074 (PMC10158647; doi:10.1162/nol_a_00074)
Supplement: Supplementary file 1 [file nol-3-3-495-s001.docx]

**Supplementary Materials**


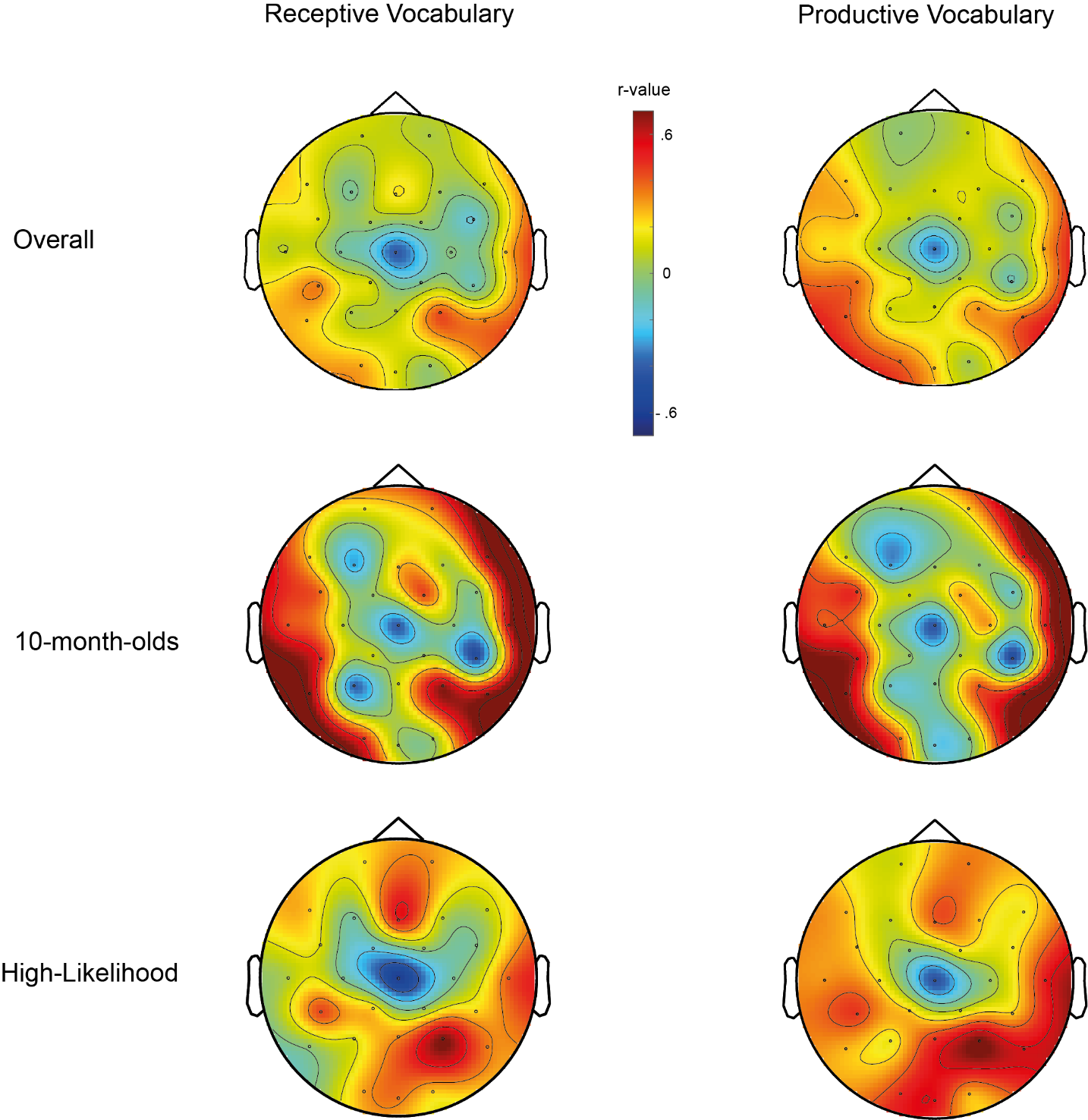


**Supplementary figure 1**

Topographic maps displaying the correlations between speech-brain coherence in the stressed syllable rate and vocabulary. Dots indicate the individual electrodes assessed in this study. Only groups showing a significant relationship between coherence and vocabulary in the analysis using electrode-averaged coherence values are displayed here.
